# Atlanta Academy of Medicine

**Published:** 1879-12

**Authors:** R. W. Westmoreland

**Affiliations:** Reporter


### Reports of Societies.

### ATLANTA ACADEMY OF MEDICINE.

R. W. WESTMORELAND, M.D., Reporter.

Atlanta, Nov. 3, 1879.

Academy met, the President, Dr. Todd, in the chair.

Dr. Bak read an essay on Metaloscopy, and gave notice that at the next meeting he would produce cases or reports illustrative of the principles announced in the essay. He remarked that he had been no little astonished at the skepticism of members of the local profession who were accounted erudite and enterprising.

Dr. Baird acknowledged to a decided skepticism on the subject, and would not deny that it was attributable, probably, to a lack of information. As it was, however, the whole thing appeared to him more as a fertile concatention of foolery than anything worthy of practical or serious consideration. The plea that not only functional but even organic lesions of the nervous centres are remedied by the simple application of little pieces of metal to the cuticular surface is, to him, superlatively absurd. He believed that the only source of benefit in such applications is through the effect upon the imagination of the patient.

Dr. W. F. Westmoreland followed with a quasi endorsement of the principles of metaloscopy. For the reason that so little is really known in regard to diseases of the nervous system, he thought physicians were warranted in accepting anything plausible as to their treatment. The facts adduced touching metaloscopy were presented by eminent authority, and were based upon actual observation and experiment prosecuted by those proficient in the business.

Dr. Bak is an enthusiastic advocate of the practice, and made some very eloquent and effective remarks in its defense.

The President looked with great incredulity upon the assertions made by the advocates of metaloscopy. He thought that the practice was analagous to the Hollman Pad quackery, *et id omne genus*.

The discussion was closed on the part of Dr. W. F. Westmoreland by presenting an illustrative case of the ready cure of apparently extreme nervous affections by simple measures. A soldier had been entirely deaf and dumb for a considerable length of time, so much so that the loudest and most sudden sounds were apparently unheard by him. Upon being thoroughly anesthetized, however, he not only recovered hearing, but speaking as well. Upon recovering from the anesthesia a good strapping perfected the cure.

Upon motion the Academy adjourned.

